# Using subjective expectations to model the neural underpinnings of proactive inhibition

**DOI:** 10.1111/ejn.14308

**Published:** 2019-01-01

**Authors:** Pascal Pas, Stefan Du Plessis, Hanna E. van den Munkhof, Thomas E. Gladwin, Matthijs Vink

**Affiliations:** ^1^ University Medical Center Utrecht Utrecht University Utrecht The Netherlands; ^2^ Department of Psychiatry Stellenbosch University Cape Town South Africa; ^3^ Max Planck Institute for Metabolism Research Cologne Germany; ^4^ University of Chichester Chichester UK; ^5^ Departments of Experimental & Developmental Psychology Utrecht University Utrecht The Netherlands

**Keywords:** behavioral control, cues, expectations, fMRI, striatum

## Abstract

Proactive inhibition – the anticipation of having to stop a response – relies on objective information contained in cue‐related contingencies in the environment, as well as on the subjective interpretation derived from these cues. To date, most studies of brain areas underlying proactive inhibition have exclusively considered the objective predictive value of environmental cues, by varying the probability of stop‐signals. However, by only taking into account the effect of different cues on brain activation, the subjective component of how cues affect behavior is ignored. We used a modified stop‐signal response task that includes a measurement for subjective expectation, to investigate the effect of this subjective interpretation. After presenting a cue indicating the probability that a stop‐signal will occur, subjects were asked whether they expected a stop‐signal to occur. Furthermore, response time was used to retrospectively model brain activation related to stop‐expectation. We found more activation during the cue period for 50% stop‐signal probability, when contrasting with 0%, in the mid and inferior frontal gyrus, inferior parietal lobe and putamen. When contrasting expected vs. unexpected trials, we found modest effects in the mid frontal gyrus, parietal, and occipital areas. With our third contrast, we modeled brain activation during the cue with trial‐by‐trial variances in response times. This yielded activation in the putamen, inferior parietal lobe, and mid frontal gyrus. Our study is the first to use the behavioral effects of proactive inhibition to identify the underlying brain regions, by employing an unbiased task‐design that temporally separates cue and response.

**Abbreviations**
IPC: Inferior parietal cortex
MRI: Magnetic resonance imaging
PMd: Dorsal pre‐motor cortex
rIFC: Right inferior frontal cortex
rIFG: Right inferior frontal gyrus
rIPC: Right inferior parietal cortex
SMA: Supplementary motor area
SSAT: Stop‐signal anticipation task
SSD: Stop‐signal delay

## 1 INTRODUCTION

Anticipating future events is a fundamental hallmark of higher‐order cognitive control, as it serves to improve performance and consequently aid survival. For example, by delaying a response, the chance that the response can be inhibited successfully or alternative action can be taken is increased (Logan & Cowan, 1984). This type of anticipation is commonly referred to as proactive inhibition (Chikazoe, Jimura, Hirose, et al., 2009; Verbruggen & Logan, 2009; Vink, Kaldewaij, Zandbelt, Pas, & du Plessis, 2015; Vink et al., 2014; Vink, de Leeuw, et al., 2015; Zandbelt, Bloemendaal, Neggers, Kahn, & Vink, 2012; Zandbelt & Vink, 2010).

Tasks that are designed to engage proactive inhibition typically use cues at the start of each trial to indicate the likelihood of a stop‐signal. We have consistently shown that reaction times increase with an increasing stop‐signal likelihood in healthy adult subjects (Vink et al., 2005), but not in children (Vink et al., 2014), the elderly (Kleerekooper et al., 2016), and various psychiatric patient groups (Vink, Ramsey, Raemaekers, & Kahn, 2006). In the brain, proactive inhibition involves activity in a network associated with stopping, consisting of the striatum, supplementary motor area (SMA), dorsal premotor cortex (PMd), right inferior frontal gyrus (rIFG), and right inferior parietal cortex (rIPC) (Vink et al., 2005; Chikazoe, Konishi, Asari, Jimura, & Miyashita, 2007; Chikazoe, Jimura, Hirose, et al., 2009; Jahfari, Stinear, Claffey, Verbruggen, & Aron, 2010; Zandbelt & Vink, 2010; Duque, Labruna, Verset, Olivier, & Ivry, 2012; Zandbelt et al., 2013; van Belle, Vink, Durston, & Zandbelt, 2014).

**Figure 1:**
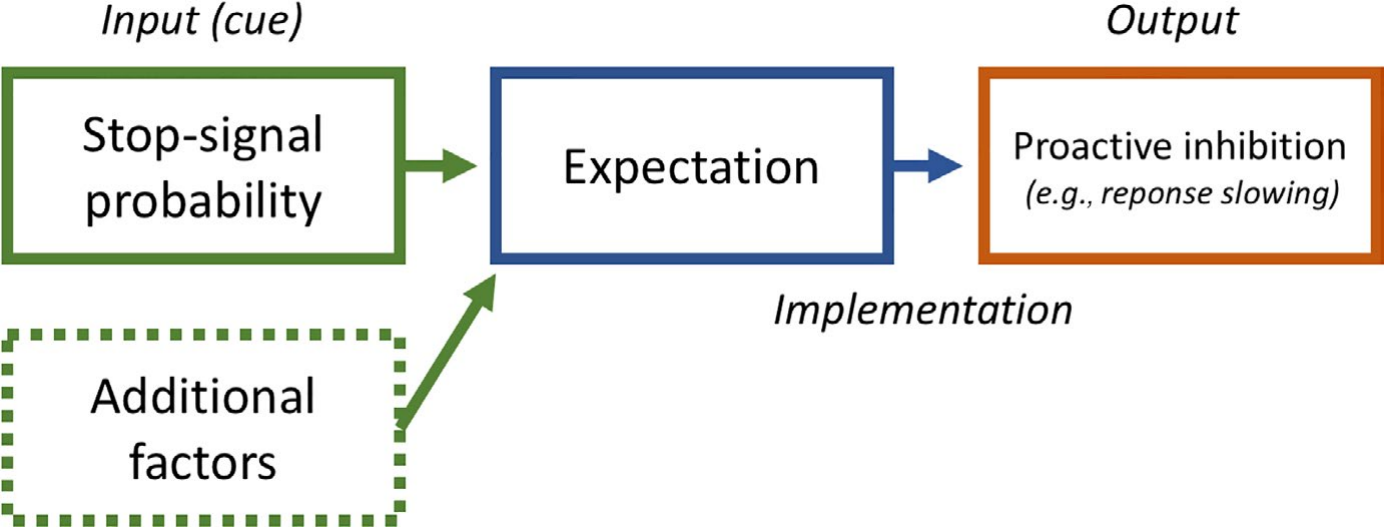
Schematic representation of the processes that lead up to proactive inhibition. In terms of a stop‐signal task, a cue represents the probability of a stop occurring. However, subjects may interpret identical cues differently, and can therefore vary in their subjective expectation on a trial‐by‐trial basis. This expectation of a stop occurring or not will subsequently lead to the slowing of responses. Our current research uses this response slowing to disentangle the processes leading up to response inhibition, and model brain activation specifically related to the expectation of having to stop. [Colour figure can be viewed at wileyonlinelibrary.com]

To date, most studies investigating the neural components of proactive inhibitory control have relied solely on modeling brain activation changes related to variations in the objective stop‐signal likelihood indicated by cues (see Figure 1 for a schematic representation). However, as we have shown (Vink, de Leeuw, et al., 2015; Vink, Kaldewaij, et al., 2015 Pas, van den Munkhof, du Plessis, & Vink, 2017) subjects’ expectations vary greatly within a stop‐signal category. We used an unbiased design in which the cue was separated in time (1,000 to 2,000 ms) from the presentation of the stimulus and subsequent response. In doing so, we are able to investigate preparatory proactive processes associated with the cue independent from the actual stimulus and response. Immediately after the presentation of the cue, which indicated stop‐signal likelihood, subjects had to indicate whether or not they expected a stop‐signal in the upcoming stimulus (Vink, de Leeuw, et al., 2015; Vink, Kaldewaij, et al., 2015). With this task, we found that while the cues objectively represented an average stop‐signal likelihood, subjects varied in their subjective expectation whether or not a stop‐signal will occur and thus in the amount of proactive inhibitory control (Zandbelt et al., 2013). Using this approach, we were able to show for the first time that activation in the striatum, SMA, PMd, and midbrain is related to the subjective expectation of having to stop a response.

However, this approach is limited because [a] it forces subjects to translate their gut feeling into a two‐choice response (‘yes’ or ‘no’), and [b] subjects have no way to indicate the level of certainty of their response. So, rather than asking subjects what they expect, we propose to use trial‐to‐trial variations in reaction times during the stimulus and response period as indicator of stop‐signal expectation already during the cue period. Some support for this approach comes from data from our previous study, in which we found that on average subjects showed the slowest responses when they indicated they expected a stop‐signal (Vink, de Leeuw, et al., 2015; Vink, Kaldewaij, et al., 2015). Since proactive inhibition is characterized by the slowing down of responses, we use this objective behavioral marker to investigate its neural underpinnings.

In our current paper, we model the amount of proactive inhibition based on the trial‐to‐trial variations in response speed for trials with a 50% stop‐signal probability. To date, several studies used reaction times to investigate inhibitory control. For example, Hu and Li (Hu & Li, 2011) showed significant contributions of the bilateral putamen, inferior parietal lobe, and right prefrontal cortex to preparatory inhibition by using a complex statistical model that, among other factors, included a contrast between fast and slow trials. Moreover, Li and colleagues have employed several intricate Bayesian models to show that prolonged response times are associated with activation in the pre‐SMA and insula (Hu, Ide, Zhang, & Li, 2015), and that the longer intervals between cue and response may disrupt proactive inhibitory control (Wang et al., 2018).

We now take a much simpler approach of investigating proactive inhibition by parametrically modeling brain activation during the cue period based on response speed during the actual stimulus and response period. This allows us to use trial‐to‐trial variations in response speed as indicator of the amount of proactive inhibitory control that is being engaged independent from the general stop‐signal probability context as indicated by the cue. Importantly, we employed a design in which brain activation during the cue period can be modeled independently from activation during the stimulus and response period (Zandbelt et al., 2013; Vink, de Leeuw, et al., 2015; Vink, Kaldewaij, et al., 2015; Pas et al., 2017). In this task, the cue period and stimulus‐response period are adequately separated in time (a delay varying between 1,000 and 2,000 ms), so that interpretation of brain activation during the cue period is not biased by response speed (e.g. longer responses allowing for greater buildup of blood oxygenation level dependency‐signal).

Here, we use functional magnetic resonance imaging (MRI) and an unbiased design to investigate the neural underpinnings of proactive inhibition by means of parametrically modeling response speed. Twenty‐five healthy volunteers performed a modified delayed‐response stop‐signal anticipation task (SSAT) while being scanned with functional MRI. During the cue period, a cue indicates the stop‐signal probability (0% or 50%), and subjects are asked whether or not they expect a stop‐signal to occur (yes/no/don('t know). After a variable delay (ranging from 1,000 to 2,000 ms), the stimulus is presented, requiring subjects to respond (go trials) or refrain from responding (stop trials). Finally, brain activation during the cue is parametrically modeled based on response times during the stimulus and response period. Proactive inhibition networks are investigated using three contrasts: the effect of stop‐signal probability (0% vs. 50%), the effect of subjective expectation (expected vs. not‐expected trials during the 50% trials), and the parametric effect of response speed during the 50% trials. In this way, we were able to investigate proactive inhibition by means of the effect of cues, subjects’ expressed subjective experience, and what people actually do. Importantly, we focused on the cue period that occurs 1,000 to 2,000 ms prior to the onset of the stimulus. By doing so, we center our analyses on preparatory processes. Given our design, these processes are not contaminated by processes underlying actual responding and feedback processes triggered by the response (Zandbelt et al., 2013). We hypothesized that by modeling activation during the cue period with subjects’ subsequent responses will more accurately highlight the brain regions associated with proactive inhibition. We will be able to remove unexplained noise that is left in our data when contrasting brain activation for the two cues, or taking into account expressed subjective experience and contrasting expected versus unexpected stop‐signals.

## 2 MATERIALS AND METHODS

### 2.1 Subjects

Twenty‐five volunteers (age *M* = 21.6 years, *SD* = 2.7; 5 females, 20 males) participated in the experiment. All subjects were right‐handed, reported no history of psychiatric or neurologic disorders and gave written informed consent. The study was approved by the ethics committee of the University Medical Center Utrecht. This study conformed to the 2013 WMA Declaration of Helsinki. The dataset was previously used to investigate brain activation during reactive inhibition (Pas et al., 2017), our current analyses deal with the neural underpinnings of proactive inhibition and are limited to the cue phase of the experiment.

### 2.2 Stop‐signal anticipation task

**Figure 2:**
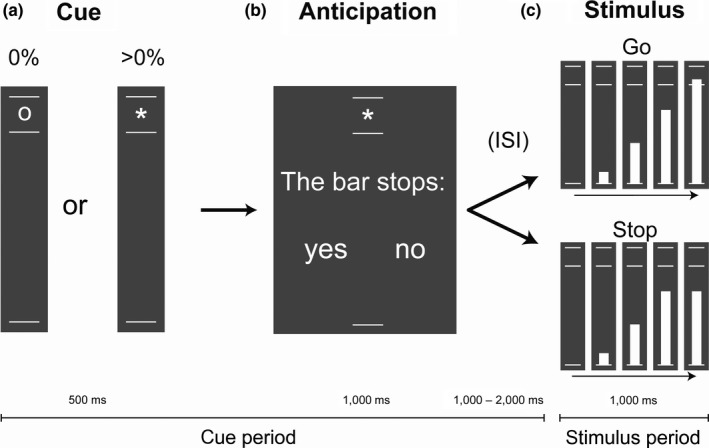
Delayed‐response stop‐signal anticipation task. On each trial, a bar moved at constant speed from the bottom line to the upper line, reaching the middle white line in 800 ms (c). The aim is to stop the moving bar as close to the middle white line as possible by pressing a button with the right thumb. These trials are referred to as go trials. In some trials, the bar stops moving automatically before reaching the middle white line (stop), indicating that subjects have to refrain from reacting. The stop‐signal delay (SSD) was initially set at 550 ms and was varied in steps of 33 ms according to a tracking procedure (SSD is increased after a successful stop trial; SSD is decreased after stop‐trials in which subjects fail to inhibit). At the beginning of each trial, the stop‐signal probability was indicated by a cue (the exact stop‐signal probability was not visible for the subjects) (a). Immediately after this cue, subjects indicated whether they expected a stop‐signal in the upcoming trial by pressing a button (yes/no) (b). They were not forced to make a decision; the task continued after 1,000 ms regardless of a response. ISI, inter‐stimulus interval. The inter‐trial interval (not pictured) varied from 1,000 to 2,000 s

Subjects performed the SSAT (Zandbelt et al., 2013), a stop‐signal task designed to measure proactive and reactive inhibitory control. The task and experimental procedures were adapted from (Vink, de Leeuw, et al., 2015; Vink, Kaldewaij, et al., 2015), see Figure 2 for an overview. In short, subjects were instructed to stop a moving bar on the screen (referred to as “go” trials). In some trials, the bar stops moving on its own (referred to as the stop‐signal) and subjects have to refrain from responding. At the beginning of each trial, a cue indicates the probability that the bar will stop: either a ‘0’ indicating no chance of a stop‐signal occurring, or a ‘*’ indicating the possibility that a stop‐signal could occur. Subjects were asked immediately following the cue to answer the question: ‘Do you expect a stop‐signal?’ by pressing a button corresponding to ‘yes’ or ‘no’. This provided us with information concerning the subjects’ subjective stop‐signal expectation. If subjects did not respond within 1,000 ms, the trial was coded as ‘don't know’. Also, if subjects had no expectation at all, they were instructed to refrain from making a choice and the trial would continue in the same fashion. Task difficulty was managed in a step‐wise fashion, with a varying delay between the stop cue and the target depending on correct or incorrect trials. This ensured overall stop accuracy to be around 50% for each individual subject. In total, 180 trials were presented, 60 trials with 0% stop‐signal probability and 120 trials with a 50% stop‐signal probability. These trials were ordered in a pseudo‐random sequence that was fixed across subjects.

### 2.3 Data acquisition

Imaging was performed on a 3.0 T Achieva whole‐body MRI scanner (Philips Medical Systems, Best, the Netherlands) at the University Medical Center Utrecht. Functional (T2*‐weighted) echo planar images with blood oxygen level‐dependent contrast oriented in a transverse plane tilted 20° over the left–right axis were obtained in a single run (683 volumes; 30 slices per volume; voxel size, 4 mm isotropic; repetition time, 1,600 ms; echo time, 23 ms). A whole brain T1‐weighted structural image (185 slices; repetition time, 8.4 ms; echo time, 3.8 ms; flip angle, 8°; field of view, 252 × 185 × 288 mm; voxel size, 1 mm isotropic) was acquired for within‐subject registration purposes.

### 2.4 Analyses

The percentage of trials was calculated where subjects expected a stop to occur. This was done separately for trials with a 0% stop‐signal probability and for trials with a 50% stop‐signal probability. In addition, the effect of stop‐signal expectation on accuracy and response times was assessed. The impact of stop‐signal expectation on the speed of inhibition was measured by the stop‐signal reaction time, computed according to the integration method (Logan & Cowan, 1984). The stop trial accuracy was also determined for both stop‐signal expectation conditions.

### 2.5 Imaging

Image data were processed using SPM8 (http://www.fil.ion.ucl.ac.uk/spm/). Preprocessing involved realignment, slice timing correction, spatial normalization to the Montreal Neurological Institute template brain, and smoothing (8 mm full width at half maximum) to correct for inter‐individual differences. Functional images were submitted to a general linear model regression analysis. Activation time‐locked to the presentation of the cue and to the stimulus response period was modeled based on stop‐signal probability and stop‐signal expectation. Trials in which a subject did not indicate an expectation, trials with zero stop‐signal probability where subjects nonetheless expected a stop and incorrect go trials were considered as errors and added to the nuisance factor. Cue‐related activation was modeled as epochs of 1,500 ms. On average the inter‐trial interval was 1,000 ms (ranging from 500 to 1,500 ms), and served as an implicit baseline. Six realignment parameters were added as regressors of no interest to correct for head motion. All data were high‐pass filtered with a cut‐off of 128 s to control for low‐frequency drifts.

To investigate the brain regions associated with proactive inhibition, we performed three whole brain analyses during the cue period. First, to investigate the effect of stop‐signal probability, we contrasted activation in the brain during go trials for cues indicating the possibility of a stop‐signal, and those without. Second, we tested the effect of expectation in go‐trials for the cues with a stop‐signal probability of 50%, by contrasting those cues where subjects expressed expecting a stop to occur, with those in which they did not. Last, we looked at brain activity for the go‐trials in which a stop‐signal could occur (i.e., within the set of 50% cue trials), with subjects’ subsequent response times included as a parametric modulator in a separate model. For this, the hemodynamic response function is convolved with a signal containing delta peaks multiplied by the response times, as we expect more brain activation during the cue period when subjects slowed down afterwards. All brain activation maps will be thresholded at a family wise error‐corrected cluster level of *p* < 0.05, with cluster sizes determined using CorrClusTh (http://www.sph.umich.edu/-nichols/JG5/CorrClusTh.m).

## 3 RESULTS

### 3.1 Behavior

An overview of the percentage of trials in which a stop‐signal was expected is presented in Table 1. Subjects expressed their expectations in accordance with the cue probability, with expected and unexpected trials differing significantly between the conditions. The amount of trials in which subjects did not express an expectation was not significantly different. Accuracy and response times are presented in Table 2. Accuracy on go trials was close to 100% for all conditions. As task‐difficulty was managed in a step‐wise fashion, overall accuracy on stop trials was as expected, with 52% (*SD* = 3). Subjects performed significantly better on trials with expected stops (*M* = 59%, *SD* = 7) than trials with unexpected stops (*M* = 46, *SD* = 9, *t*(25) = 4.2, *p* < 0.001). Bonferroni's correction for multiple comparisons was used to adjust the significance level.

**Table 1:** Stop‐signal anticipation per trial type as mean (±*SD*) percentage of trials

	0% stop‐signal probability	>50% stop‐signal probability	Paired samples *t*‐test
Stop‐signal expected	2 ± 5	48 ± 14	*t*(24) = −14.3, *p* < 0.001
Stop‐signal not expected	95 ± 5	45 ± 12	*t*(24) = 16.9, *p* < 0.001
No expectation indicated	3 ± 2	7 ± 14	*t*(24) = −1.8, *p* = 0.09

**Table 2:** Mean (±*SD*) accuracy and response times per trial type

Trial type	0% stop‐signal probability	50% stop‐signal probability			*p*‐value
		Not expected	Expected	Test value	
Go					
Accuracy (%)	95 ± 5	98 ± 3	97 ± 4	*F*_2,23_ = 3.6	0.044
RT (ms)	807 ± 17	845 ± 26	857 ± 24	*F*_2,23_ = 57.6	<0.001
Stop					
Accuracy (%)		46 ± 9	59 ± 7	*t*(24) = 4.2	<0.001
SSRT (ms)		231 ± 18	226 ± 22	*t*(24) = 1.1	0.28

**Figure 3:**
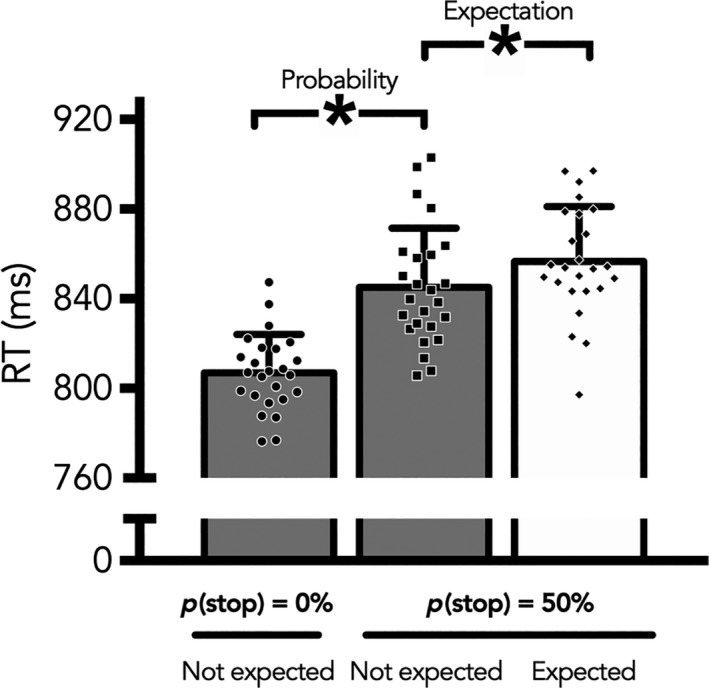
Response times for all conditions. Subjects were slower on trials with a stop‐signal probability of 50%, and even slower when they also expected one to occur. *p < 0.001

In line with previous findings (Zandbelt et al., 2013; Vink, de Leeuw, et al., 2015; Vink, Kaldewaij, et al., 2015) subjects responded more slowly on trials where a stop‐signal could occur *t*(24) = 10.1, *p* < 0.001. When we only included trials where subjects did not expect a stop, subjects were again slower on trials where a stop could occur *t*(24) = 9.6, *p* < 0.001. Finally, when subjects expected a stop, their responses slowed down even more *t*(24) = 3.7, *p* = 0.001. This effect of response slowing is visible in Figure 3.

### 3.2 Imaging

See our supplemental materials for a replication of our previous study (Vink, de Leeuw, et al., 2015; Vink, Kaldewaij, et al., 2015), using pre‐defined regions of interest.

**Figure 4:**
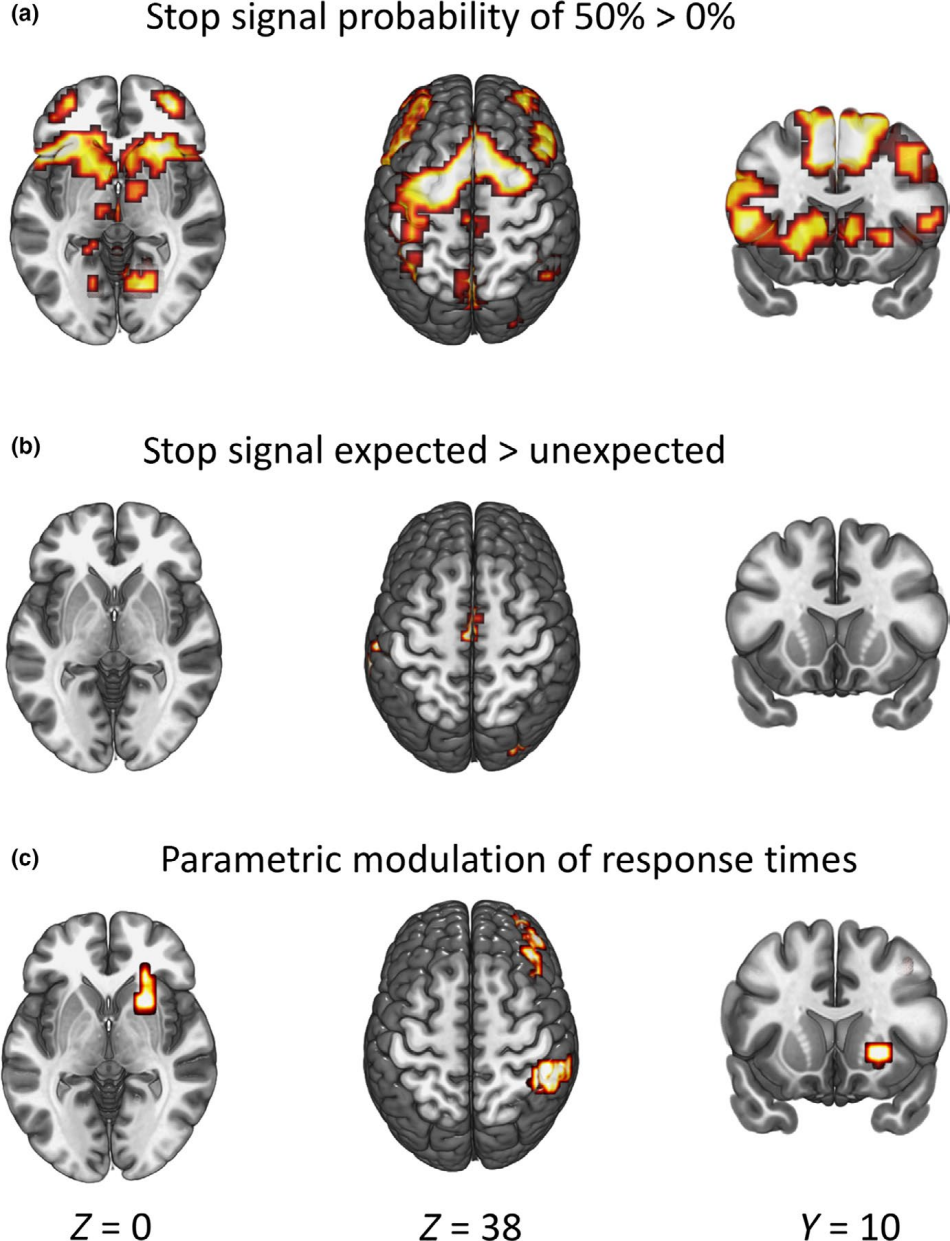
Imaging results for the three whole‐brain analyses. All brain activation maps are thresholded at a family wise error‐corrected cluster level of *p* < 0.05 (height threshold of T = 3.4). For details see Table 3. (a) Brain activation for the contrast of trials with a stop‐signal probability of 50% vs. 0%. (b) Brain activation for the contrast of the cues with a stop‐signal probability of 50% where subjects expected one to occur vs. where they did not. (c) Brain activation during the cue period modulated by subsequent response times. [Colour figure can be viewed at wileyonlinelibrary.com]

When contrasting the cues indicating a stop‐signal probability of 50% with those indicating 0% stop‐signal probability, we found activation in mid and inferior frontal gyrus, inferior parietal lobe and putamen (Figure 3a), indicating that these areas are involved with the possible inhibition of a future response or the processing of environmental cues. Our second analyses contrasted the cues with a stop‐signal probability where subjects expected a stop, with those in which they did not expect one. This analysis yielded again significant activation in the mid frontal gyrus, parietal lobe and in the occipital gyrus (Figure 4b). Finally, we modeled activation during the cue period using subsequent response time as a parametric modulator. We found that activation in the mid frontal gyrus, inferior parietal lobe and right putamen positively correlated with response time (Figure 3c). Analogous to our model depicted in Figure 1, the results from Figure 3a only take into account the input of the stop‐signal probability cues, Figure 3b uses a contrast based on subjects’ expressed subjective experience, and the results from Figure 3c are based on the behavioral output – i.e. response slowing. See table 3 for an overview of activation clusters.

## 4 DISCUSSION

Here, we used functional MRI and an unbiased design to investigate the neural underpinnings of proactive inhibition by means of parametrically modeling response speed to the cue period of a stop‐signal response task. In this task, cues were presented 1,000–2,000 prior to the onset of the stimulus and response period. Cues indicated stop‐signal probability (0%, 50%) and subjects indicated whether or not they expected a stop‐signal. Actual response speed during the stimulus and response period was taken as an indicator of subjective expectation of a stop‐signal (Figure 4; Table 3).

**Table 3:** Overview of activations

Region	BA	Side	No of voxels	X	Y	Z	Max *t*‐value
(A) 50% > 0% probability cues							
Mid. Frontal gyrus	6/8	L/R	2,390	−4	24	44	9
Inf. Frontal gyrus, putamen	47	R	132	28	24	−4	6.49
Precuneus	7	L/R	163	−4	−64	48	5.29
Inf. Parietal lobe	40	R	101	48	−48	40	5.15
(B) Expected > unexpected							
Occipital gyrus	19	R	46	32	−84	16	5.34
Mid. frontal gyrus	6	L/R	51	0	−4	48	5.13
Inf. Parietal lobe	40	L	36	−56	−24	32	4.48
(C) Modulation of response times							
Putamen, Inf. frontal gyrus	47	R	33	24	12	−4	5.27
Inf. Parietal lobe	40	R	132	52	−44	56	5.27
Mid. Frontal gyrus	10	R	95	40	48	24	4.81

*Note*. All results are significant at a family‐wise error corrected cluster level of *p* < 0.05; L, left; R, right; X Y Z refer to the center of mass.

We found significantly more activation during the cue period for 50% stop‐signal probability, when contrasting with 0%, in the right putamen, inferior and mid frontal gyrus and inferior parietal lobe. When contrasting expected vs. unexpected trials, we found modest effects in the mid frontal gyrus, parietal lobe, and in the occipital gyrus. With our third contrast, we modeled brain activation during the cue with trial‐by‐trial variances in response times. This yielded significant results in the putamen, inferior parietal lobe, and mid frontal gyrus. By using response slowing as an indication of the expectation of having to stop, that is only partially modulated by the cues, we were able to obtain a more precise estimate of the role brain regions have in sub serving proactive inhibition.

Activation of the putamen during cues with 50% stop probability and when responses were slower is in line with our previous research showing that activation in the striatum, of which the putamen forms part, depended on subjective anticipation of stop‐signals, in the cue phase (Vink, de Leeuw, et al., 2015; Vink, Kaldewaij, et al., 2015) and during reactive inhibition (Pas et al., 2017). Similarly, Hu and Li (2011) found bilateral activation in the putamen when looking at anticipatory motor preparation. However, this research looked at a more general type of motor preparation that was not specifically modeled to take into account trial‐by‐trial variations in response times.

Striatal activation during reactive inhibition may be part of the same process involved in striatal activation during stop‐signal anticipation. In the case of successful inhibition, a stop‐signal might have been anticipated already at the onset of the trial. In order to successfully inhibit a response, one simply needs to refrain from responding, without the need for active inhibition, if no response was prepared or initiated in the first place. Indeed, response inhibition studies have commonly reported striatal activity in anticipation of a highly predictable stop‐signal (Aron & Poldrack, 2006; Vink et al., 2005, 2006; Vink, de Leeuw, et al., 2015; Vink, Kaldewaij, et al., 2015Zandbelt, van Buuren, Kahn, & Vink, 2011; Zandbelt & Vink, 2010).

Our results are supported by studies implicating the striatum in the control over actions (Kimura, 1992; Chen, Scangos, & Stuphorn, 2010; Watanabe & Munoz, 2010; Duque et al., 2012; Zandbelt et al., 2013). Functionally, the putamen is closely connected with the motor cortex (Duann, Ide, Luo, & Li, 2009; Forstmann et al., 2008; Vergani et al., 2014; Vink et al., 2005; Zandbelt & Vink, 2010), and consequently involved in basic motor inhibition and response switching (Forstmann et al., 2008). In terms of its role in proactive inhibition, Hu and Li (2011) found activation in the putamen to be specifically linked to preparatory motor execution. The broader area of the basal ganglia have been hypothesized to act as a gatekeeper, preventing execution of conflicting motor responses (Friend & Kravitz, 2014; Mink, 1996). A more overarching role of the striatum is likely the selection of responses, and the inhibition of unselected responses, based on prior reinforcement (Vink, Pas, Bijleveld, Custers, & Gladwin, 2013).

Our current results show that this area, specifically the putamen, is involved in the process of proactive inhibition, and linked to the anticipation of stop‐signals. Indeed, striatal activity has been linked to the expectation of higher effort demands (Pas, Custers, Bijleveld, & Vink, 2014). Activation has also been demonstrated to increase during cue‐learning paradigms, with the region being linked to the formation of stimulus‐response associations (Diederen, Spencer, Vestergaard, Fletcher, & Schultz, 2016; Vink et al., 2013). During our task, subjects constantly have to ascribe a subjective weight to the cue they are given – what do they actually believe is going to happen. This can therefore be seen as comparable to the learning phase of a cue‐learning paradigm. Tricomi, Delgado, McCandliss, McClelland, and Fiez (2006) showed that striatal activation was linked to the incorporation of feedback in a learning task, and data by Seger (2005) reaffirm its role in identifying the behavioral context for selection of an appropriate strategy. Striatal contributions to proactive inhibition could therefore lie in selecting the optimum response and linking cues with the appropriate behavior.

When contrasting 50% over 0% stop probability, we found elevated activity in the inferior and mid frontal gyrus (IFG). In addition to the striatum, the right inferior frontal gyrus (IFG) has long been recognized as playing an important role in proactive inhibition (Aron, Fletcher, Bullmore, Sahakian, & Robbins, 2003; Rubia, Smith, Brammer, & Taylor, 2003; Vink, de Leeuw, et al., 2015; Vink, Kaldewaij, et al., 2015). An increase in functional connectivity between this area and the basal ganglia has been shown to increase response inhibition efficiency (Xu et al., 2016) and rIFG activity has been correlated with stopping speed (Whelan et al., 2012). In contrast, hypoactivation of the rIFG in patients with ADHD has been linked to impaired response inhibition (Morein‐Zamir et al., 2014). However, there remains controversy as to whether the rIFG is involved in stopping directly or in attentional engagement necessary for response inhibition, due to opposing findings and paradigmatical problems. A prominent line of reasoning is that this region is critical in the act of general stopping (Aron, 2011), functioning as a breaking mechanism that can either lead to outright stopping, or to a pausing or slowing down of responses (Aron, Robbins, & Poldrack, 2014; Cai, Ryali, Chen, Li, & Menon, 2014). Aron et al. (2014) argue that rIFG activation represents a brake which is a form of partial stopping and does not necessarily lead to actual stopping. In this sense, cue‐related rIFG activation during an unsuccessful stop trial can still represent the triggering of a stopping response, instead of representing attention only. In line with this, the rIFG is also activated by an internal motivation to stop in the absence of external cues (Brass & Haggard, 2007). Yet Hampshire and colleagues (Hampshire, 2015; Sharp et al., 2010) propose the alternative view that rIFG recruitment is related to detection of important cues, instead of to the subsequent suppression of motor responses. Indeed, cognitive control was primarily engaged for contextual cue monitoring instead of the actual stopping, during a response inhibition task (Chatham et al., 2012) and the rIFC directs attentional processes (Baldauf & Desimone, 2014). Findings from the psychiatric field support this theory as well. For example, in patients suffering from post‐traumatic stress disorder, rIFG functioning has been directly linked to the processing of contextual cues (van Rooij et al., 2014), while in schizophrenia patients reduced activation in the rIFG and temporoparietal junction was accompanied by impairments in the processing of cues aiding proactive inhibition (Zandbelt et al., 2011). Therefore, involvement of the rIFG in cue processing might be mediated by increased attention to these cues. Inherent to most tasks, it is impossible to completely distinguish attentive and inhibitory processes. Though Boehler, Appelbaum, Krebs, Chen, and Woldorff (2011) showed that the rIFG only responded to relevant stop cues and not to irrelevant stop cues appearing in a control block in which the subject was instructed to ignore the cues, pointing to a role in inhibition and not to infrequent cue detection, it might still be related to attentional engagement. It might also be that differential roles in attention and inhibition are regulated by distinct subareas within the rIFG or by differential network involvement (Chikazoe, Jimura, Asari, et al., 2009; Sebastian et al., 2016; Verbruggen, Aron, Stevens, & Chambers, 2010). Distinct functional roles in response inhibition have already been assigned to different IFG subregions (Cai & Leung, 2011; Chikazoe, Jimura, Asari, et al., 2009). Alternatively, the rIFG might be generally involved in response control instead of in inhibition per se. Indeed, in a Go‐NoGo task, Dodds, Morein‐Zamir, and Robbins (2011) reported strong rIFG activation during a third cue type that instructed subjects to press an additional button. Although its exact role remains unclear, taken together with our results, this places the rIFG in a multiple demand network (Kolodny, Mevorach, & Shalev, 2017).

Activity in the mid frontal gyrus correlated with increasing response time, as an objective measure for stop‐signal expectation. The dorsolateral prefrontal cortex (DLPFC), a prominent structure within the mid frontal gyrus, has been associated with response inhibition before (Hege, Preissl, & Stingl, 2014; Hung, Gaillard, Yarmak, & Arsalidou, 2018; Luijten et al., 2014) and is thought to be involved in executive function, cognitive flexibility, and planning. Specifically, this area was more active during conditional stopping ‐when subjects only had to stop their responses in a specific context‐ than during simple stopping (Chikazoe, Jimura, Hirose, et al., 2009; Swann et al., 2009) and is also though to implement task rules (Aron et al., 2014). Possibly in our task, when slowing down more in expectation of a stop‐signal, subjects are planning a change in response, which requires more cognitive control and flexibility than during unexpected stop trials, which is reflected in enhanced activity in the mid frontal gyrus. In all three contrasts we found increased activation in the inferior parietal cortex (IPC). Activity in the right IPC has been linked to self‐initiated as opposed to triggered or automatic responses (Kühn, Haggard, & Brass, 2009). Kühn and colleagues suggest that this region plays a role in inhibitory processes when voluntary suppression of a response requires more selection effort or attention. Other research has linked the right inferior parietal cortex (rIPC) to the storage of acquired motor skills (Niessen, Fink, & Weiss, 2014), as lesions to this region disrupt the ability to perform previously learned actions (Halsband, Schmitt, Weyers, & Binkofski, 2001), and has demonstrated its involvement in response selection processes (Dippel & Beste, 2015). Together with our findings, this suggests that the rIPC could be involved in the decision for an alternative motor response when the probability or expectation of having to change the response, e.g., on encountering a stop‐signal, is high.

## 5 LIMITATIONS

Our task repeatedly asks subjects whether they expect a stop‐signal or not, based on a single cue. It may very well be that subjects’ reported expectation is not fully correlated with their internal subjective expectation. However, it is not necessarily relevant whether our self‐report measurement was actually able to capture our subjects’ expectations, nor whether choosing ‘yes’ or ‘no’ steered their behavior accordingly. The objective of our task was to capture subjects’ subjective expectation of having to inhibit a response and linking that expectation to neural activity. Nevertheless, the self‐report appears to have face validity and our results show an effect of this reported expectation on both behavioral measurement and in brain activation. By using response time as a parametric modulator of brain activation during the preceding cue period, we have also included an objective index of proactive inhibition.

Our results are based on the assumption that the slowing down of responses can, at least partially, be explained by the expectation people form based on the cues. However, a multitude of factors come into play on each trial that can affect response times, ranging from the effectiveness and speed of sensory processing, quality of information processing or a potential a priori bias for a specific response. Sequential‐sampling models like the diffusion model describe decision‐making as a process of noisy accumulation of evidence from a stimulus (Ratcliff, Smith, Brown, & McKoon, 2016). Reaction time variability can therefore not be claimed to exclusively depend on the subjective expectation of a stop signal, and there is still noise left unexplained.

## 6 CONCLUSION

Proactive inhibition is the slowing down of behavioral responses just before a possible full stop. We have shown that this concept cannot be fully investigated by looking only at the processing of objective cue information, but that it is necessary to take into account the variability in the effect that cues have on behavior. Paradigms solely relying on objective information derived from cues to investigate proactive inhibition are missing an important factor when interpreting the results, namely how those cues are processed and interpreted by the individual at that point in time. With our current experiment, we have used this behavioral component to demonstrate that activation in the putamen, mid frontal cortex, and inferior parietal cortex were related to the expectation of having to inhibit a response, i.e., proactive inhibition. These results allow us to build towards a more complete model of response inhibition, delineating the roles of objective and subjective information.

## CONFLICT OF INTEREST

None of the authors have any conflicts of interest.

## AUTHORS’ CONTRIBUTIONS

Pascal Pas: Experimental design, writing manuscript, data collection, data analysis. Stefan Du Plessis: Experimental design, writing manuscript. Hanna van den Munkhof: Writing manuscript, data collection, data analysis. Thomas Gladwin: Writing manuscript, data analysis. Matthijs Vink: Experimental design, writing manuscript, data analysis.

## Data Availability

The datasets analysed during the current study are available from the corresponding author (at p.pas@umcutrecht.nl) on reasonable request.
