# Supplementary material for: Using subjective expectations to model the neural underpinnings of proactive inhibition
Source: Eur J Neurosci. 2019 Jan 1;49(12):1575–86. doi: 10.1111/ejn.14308 (PMC6618303; doi:10.1111/ejn.14308)
Supplement: Supplementary file 2 [file EJN-49-1575-s002.pdf]

Supplemental materials

The following serves as a replication analysis of our previous research (Vink *et al.*, 2015). Here, our task was set up with multiple levels of stop-signal probability, and therefore the level of subjective expectation was related to objective stop-signal probability. The task contained two levels of stop-signal probability (25% and 34%), and subjects indicated that they expected a stop-signal in 25% and 75% of the trials, respectively. Although we grouped together the two levels of stop-signal probability to minimize the impact of the potential confound, these results needed to be replicated in an unbiased design. Therefore, we now used a single stop-signal probability level of 50% to allow the unbiased disentangling of brain activations related to subjective expectations and the processing of objective stop-signal probabilities.

To replicate our previous results, we investigated the effect of stop-signal probability and expectation during the cue and stimulus-response period in predefined regions of interest (ROIs). These ROIs were based on activation patterns from a previous study in which sample of healthy participants performed the delayed-response stop-signal anticipation task (Zandbelt et al., 2013b). For the cue period, we looked at the striatum, SMA, left PMd and midbrain. For the stimulus-response period the ROIs included the rIFG and rIPC. Mean activation level, expressed as percentage of signal change, was calculated per participant for each ROI. Identical to the previous study, paired-sampled t-tests were used to investigate differences in activation between conditions.

Imaging data

Cue period

Figure 5 shows activation in the ROIs during the cue period. Activation in the striatum $t(24) = 3.7$; $p = 0.001$, SMA $t(24) = 5.4$; $p < 0.001$ and PMd $t(24) = 5.3$; $p < 0.001$ was higher in trials with 50% stop-signal probability compared to trials with 0% probability, regardless of expectation. No such effect was observed in the midbrain region $t(24) = 1.739$; $p = 0.095$.

Next, we looked at activation in these regions for trials with 0% vs. 50% stop-signal probability while subjects did not expect a stop-signal. These analyses showed a significant difference in activation for the striatum $t(24) = -2.4$; $p = 0.022$, SMA $t(24) = -4.2$; $p < 0.001$ and PMd $t(24) = -3.0$; $p < 0.01$. Again, no difference was found in the midbrain region $t(24) = -1.2$; $p = 0.30$.

Finally, we looked at the difference in trials where subjects did versus did not expect a stop-signal to occur, in the context of 50% probability. For trials in which subjects expected a stop to occur, activity in the striatum $t(24) = -2.2$; $p = 0.035$, SMA $t(24) = -2.3$; $p = 0.028$ and PMd $t(24) = -3.9$; $p < 0.001$ was higher than when they did not expect one. Again, there were no significant differences in the midbrain region $t(24) = 1.1$; $p = 0.27$.

Stimulus and response period

Figure 6 shows activations in the ROIs during the stimulus and response period. Similar to previous studies (Zandbelt *et al.*, 2013; Vink *et al.*, 2015), we found heightened activation in both the rIFG $t(24) = 4.5$; $p < 0.001$ and rIPC $t(22) = 6.6$; $p < 0.001$ when stop-signal probability was 50% compared to 0%, regardless of stop-signal expectation.

In addition, we examined the effect of stop-signal expectation by examining a subset of trials where subjects indicated not expecting a stop-signal. There was significantly more activation in both the rIFG $t(24) = -4.2$; $p < 0.001$ and rIPC $t(22) = -6.3$; $p < 0.001$ in trials with 50% compared to 0% stop probability. Finally, we examined the effect of subjective expectation in the context of 50% stop-signal probability. There was no significant effect for stop-signal expectation on activation in the rIFG $t(24) = 1.2$; $p = 0.23$ and rIPC $t(22) = -0.4$; $p = 0.68$.

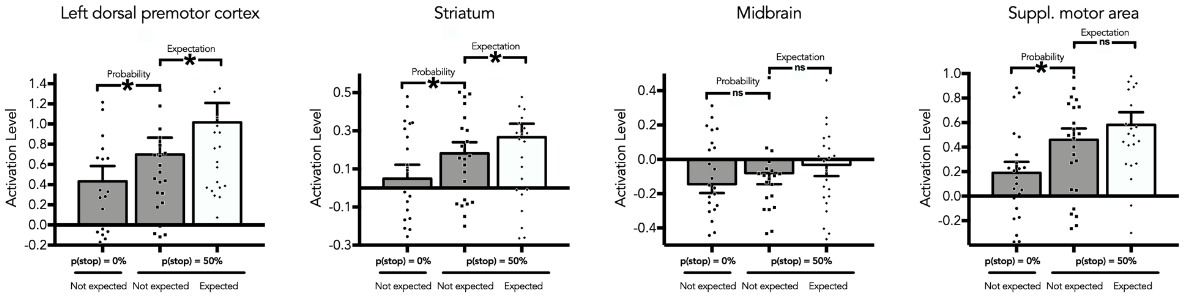

Figure 5. Activation as percent signal change in the selected ROIs during the cue period across conditions. Center coordinates for left PMd -24,10,54; Striatum -8,12,0; Midbrain -5,-31,-18 and SMA -8,5,50. *P < 0.05.

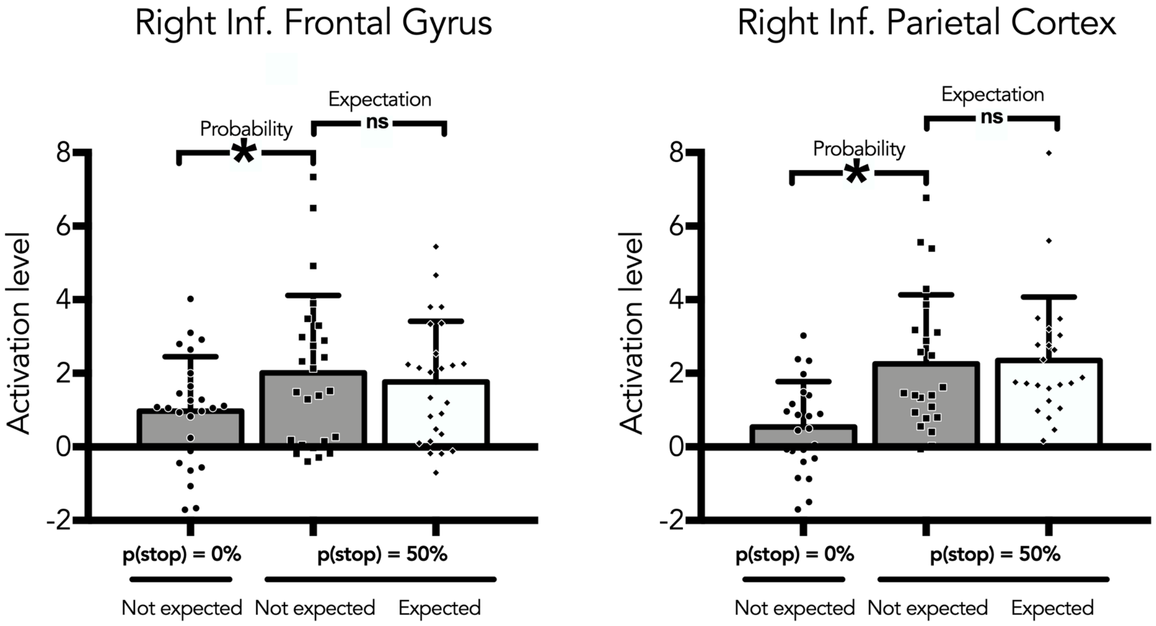

Figure 6. Activation as percent signal change in the selected ROIs during the response period across conditions. Center coordinates for rIFG 50,10,29, and rIPC 59,-40,30. *P < 0.05.
