# Supplementary material for: Using subjective expectations to model the neural underpinnings of proactive inhibition
Source: Eur J Neurosci. 2019 Jan 1;49(12):1575–86. doi: 10.1111/ejn.14308 (PMC6618303; doi:10.1111/ejn.14308)
Supplement: Supplementary file 1 [file EJN-49-1575-s001.pdf]

EJN European Journal of Neuroscience FENS Federation of European Neuroscience Societies

Using subjective expectations to model the neural underpinnings of proactive inhibition

Pas, Pascal; du Plessis, Stefan; Van den Munkhof, Hanna; Gladwin, Thomas; Vink, Matthijs

---

Review timeline:

Submission date: 12-Jul-18
Editorial Decision: Major Revision
Revision Received: 02-Nov-18
Editorial Decision: Minor Revision
Revision Received: 26-Nov-18
Accepted: 30-Nov-18

Editor: Chandan Vaidya
Reviewer 1: Alexandra Sebastian

1st Editorial Decision 28-Aug-18

Dear Dr. Pas,

First of all, apologies for the time it has taken to deal with your manuscript, this was due to difficulty in engaging reviewers. It has now been reviewed by one external reviewer as well as by the Section Editor, Dr. Chandan Vaidya, and ourselves.

The general feeling is that your paper addresses a question of broad interest using an elegant experimental design. The reviewer raises some substantive methodological issues including examination of the appropriate contrast for objective probability as well as requests more details on imaging procedures, analysis, and behavioral analysis. They also provide some suggestions for the discussion. We expect that you will be able to address the concerns and provide the requested details. Please carefully address each of the reviewer's points in a revised version of the manuscript.

In addition we noted the following points that should be addressed in the revision.
- Figure legends need to be part of the main text.
- We will need a graphical abstract/text at this stage (see below).
- Remove the section numbering.
- Include a CoI statement and list of abbreviations.
- Please include magnitude of changes in the text.
- In accordance with EJN policy please replace bar charts with more informative scatter plots, hybrid plots or similar.

When revising the manuscript, please embolden or underline major changes to the text so they are easily identifiable and please don't leave 'track change' formatting marks in your paper. Please ensure that you provide a text and a figure file for the Graphical Abstract (as detailed in the instructions below). When carrying out your revisions please refer to the checklist below and visit the EJN author guidelines at www.ejneuroscience.org

When finalized, please upload your complete revised manuscript onto the website, as a Word file (.doc, or .docx). Please also ensure that a complete set of tables and figures is included as separate files, even if these have not changed from the originals. At this stage it is necessary to provide high resolution figures.

EJN European Journal of Neuroscience FENS Federation of European Neuroscience Societies

Please see important instructions below.

Please go into https://mc.manuscriptcentral.com/ejn - Author Centre - manuscripts with decisions where you will find a 'create a revision' link under 'actions'. We ask that you please indicate the way in which you have responded to the points raised by the Editors and Reviewers in a letter. Please upload this response letter as a separate Word (.doc or PDF) file using the file designation "Authors' Response to Reviewers" when uploading your manuscript files. Please DO NOT submit your revised manuscript as a new one. Also, please note that only the Author who submitted the original version of the manuscript should submit a revised version.

If you are able to respond fully to the points raised, we would be pleased to receive a revision of your paper within 12 weeks.

Thank you for submitting your work to EJN.

Best wishes,

Paul Bolam & John Foxe
co-Editors in Chief, EJN

Reviews:

Reviewer: 1

Comments to the Author
In the present study, Pas and colleagues assess neural underpinnings of proactive inhibition with focus on objective probability and subjective expectations. To this end, 25 healthy individuals completed a modified stop anticipation task during fMRI. Each trial comprised a cue indicating the probability of a stop signal to occur (0% vs 50%), a phase in which the participants were asked about their subjective expectancy of a stop signal to occur (yes/no/don't know) and finally the stimulus presentation. The authors use data from a previous study (Pas et al., 2017, Neuroscience) but focused on the cue phase only in the current study. They used three contrasts to distinguish between the objective effect of stop signal probability on proactive inhibition (0% vs. 50% as indicated by the cue), the effect of the expressed subjective expectation (expected vs. non-expected trials during the 50% trials only), and the behavioral effect of subjective expectation as assessed by parametric modulating the cue period of the 50% trials with response speed during the stimulus phase. The rationale behind that is that the more proactive inhibition is present, the more responses are slowed. The authors report activation in right striatum, right frontal cortex and right inferior parietal cortex to the subjective expectation of a stop-signal.
Pas and colleagues use a simple and straight forward approach to study neural correlates of objective vs. subjective expectations in a proactive response inhibition task. The research question is appealing and timely, the sample size is acceptable, the methods are by and large sound and the manuscript is overall well written. Yet, I have a couple of concerns including some critical methodological issues which I outline below and which in my opinion preclude publication of the manuscript in its current form.

1.) The authors state in the abstract that they investigated "the effects of both objective probability and subjective interpretation" of stop cues. In the discussion, however, they focus on subjective expectancy

only. In addition, I believe that none of the contrast specified in the current version of the manuscript is suitable to investigate neural underpinnings of the objective probability of stop cues. The contrast “50% vs. 0%” comprises trials, in which stop-signals were subjectively expected to occur, as well as trials, in which stop-signals were subjectively expected not to occur. Therefore, this contrast of “objective probability” is contaminated by “subjective probability”. Neural underpinnings of objective probability might be assessed by contrasting “50% vs. 0%” > “stop trials in which stop signal was subjectively expected”. After having specified an appropriate contrast, the authors should focus in their discussion also on neural correlates of objective, cue-related activity.

2.) Several aspects in the methods section, in particular in the subsection '2.5 Imaging' a described imprecisely.
a) Could the authors provide more information about which SPM version has been used?
b) Was realignment really not performed in the preprocessing? It is not listed among the preprocessing steps. Since data was acquired in one single run with a duration of more than 18 minutes (683 volumes à 1600ms) (please cf. 2.3 Data acquisition), this is more than critical. Usually, a singly fMRI run has a duration of 5 to max. 10 minutes to minimize the probability of movement artifacts. Have the data been checked for movement artifacts? If so, the author should report that.
c) Stimulus-related activation is described to be modeled as an epoch of 1000ms, yet specifications about cue-related activation (when not parametrically modulated) are lacking. Was cue-related activation (when not parametrically modulated) modeled using stick functions?
d) Why was stimulus-related activation modeled as an epoch of 1000ms?
My major concern regarding the methodological approach, however, pertains to the clusterwise inference. As I gather from table 3 results were reported to be significant at a family-wise error corrected cluster level of $p < .05$. First of all, this needs to be reported in the methods section. More importantly, however, familywise error rate of 5% for clusterwise inference has been deemed invalid as they tend to result in high false positive error rates (Eklund et al., 2016, PNAS). The authors should accordingly use a more conservative approach to correct for multiple comparisons (i.e., voxelwise inference).

3.) Similar to the analyses of the fMRI data, the analyses of the behavioral are insufficiently described in the methods section. The authors vaguely describe in '2.4 Analyses' that “the effect of stop-signal expectation on accuracy and response times was assessed”. In the results sections and in table 1, the authors then present a number of paired t-tests comparing accuracy between trials with '0% stop-signal probability' and trials with '>50% stop-signal probability' (why >50%? Stop signal probability has previously been reported to be 50%?!) for the following three conditions: 1) 'stop signal expected', 2) 'stop signal not expected', 3) 'no expectation indicated'. The authors should perform a Bonferroni correction for the number of tests performed (i.e., the three conditions) or use a repeated measures ANOVA).
In addition, in the second paragraph of '3.1 Behavior', the authors should describe the comparisons more precisely. If I understand correctly, in the first t-test, go RT was compared for 0% vs. 50% trials; in the second t-test, go RT was compared for 0% vs. 50% trials on which no stop signal was expected; and in the third t-test. Go RT was compared for 50% trials on which a stop signal was expected vs. those on which no stop signal was expected? More explicit guidance might be helpful for the reader and increase clarity of the manuscript.

4.) As I gather from table 3 and Figure 4, in particular the putamen was associated with subjective expectation of a stop-signal, as revealed by with parametric modulation approach. In the text the authors report striatal activity to be associated with subjective expectation throughout the manuscript. I think it would be more specific to report putamen activity also in the text, since caudate seems not to be activated

in the given contrast. In addition, the cluster covering putamen activity as revealed by the parametric modulation approach also covered right IFG – this should also be reported in the text.

5.) The authors discuss the role of the right IFG. Hereby consideration must be given to regional specificity of the functional involvement of this rather large brain region (see e.g. the reply by Aron et al., 2014 TiCS and their criticism regarding the work of Adam Hampshire and David J. Sharp). In addition, when discussing rIFG involvement in attentional capture as reported by Sharp et al. (2010, PNAS), the authors should also mention that a number of studies using similar task reported opposing results (e.g., Boehler et al. 2011 PLoS ONE; Cai et al. 2011 PLoS ONE; Chikazoe et al. 2009 Cereb Cortex; Sebastian et al., 2016 Brain Struct Funct).

6.) The role of the middle frontal gyrus (DLPFC) in subjective expectation of a stop signal is barely discussed, although it is implicated by the parametric modulation approach.

7.) The methods within the supplemental material are insufficiently described, too. The authors should at least provide information about the nature of the ROIs (were anatomically masks used?), about the procedure of signal extraction (was mean signal activity extracted or the activity within a peak voxel? Was percent signal change extracted, or beta or contrasts estimates?) and about the test that were performed (were that rm ANOVAS? Are the results corrected for multiple comparisons?).
More information should also be provided for the supplemental figures (figures 5 and 6), at least the coordinates of the ROIs and the nature of the signal (i.e., percent signal change, beta or contrast estimates?).

Minors

i) I am sometimes confused about the tenses that the authors use. Everything pertaining to what was done in the present study should be written in the simple past tense. The authors should check the manuscript and correct the tense where applicable.

ii) '2.2 Stop-signal Anticipation task' – second sentence: " […] adapted from (Vink, Kaldewaij, et al., 2015)": please correct

iii) '2.4 Analyses' – second last sentence: "[…] stop-signal reaction time, computed according to the integration (Logan & Cowan, 1984)": should be "integration method"
iv) Discussion, p. 13, last paragraph: "Activation has also been demonstrated to increase during cue-learning paradigms; being linked to the formation of stimulus-response associations": please correct

v) Discussion, p. 14, first paragraph: "[…] and data by Seger (Seger, 2005)": should be "[…] and data by Seger (2005)"

vi) References: The publication year of the reference by Sebastian et al. should be 2016 instead of 2015.

vii) Table 3: The authors should double check the coordinates for the "Med. Frontal gyrus" (x=40 y=48 z=24) – or do they mean middle frontal gyrus instead of medial frontal gyrus?

References
Aron AR et al. (2015). Evidence Supports Specific Braking Function for Inferior PFC. Trends Cogn Sci.

19:711-712.
Boehler CN et al. (2011). The role of stimulus salience and attentional capture across the neural hierarchy in a stop-signal task. PLoS One 6:e26386.
Cai W et al. (2011). Rule-guided executive control of response inhibition: functional topography of the inferior frontal cortex. PLoS One 6:e20840.
Chikazoe J et al. (2009). Functional dissociation in right inferior frontal cortex during performance of go/no-go task. Cereb Cortex 19:146–152.
Eklund et al. (2006). Cluster failure: Why fMRI inferences for spatial extent have inflated false-positive rates. Proc Natl Acad Sci U S A. 113:7900-7905.
Sharp DJ et al. (2010). Distinct frontal systems for response inhibition, attentional capture, and error processing. Proc. Natl. Acad. Sci. U.S.A, 107:6106–6111.
Sebastian A et al. (2016). Dissociable attentional and inhibitory networks of dorsal and ventral areas of the right inferior frontal cortex: a combined task-specific and coordinate-based meta-analytic fMRI study. Brain Struct Funct 221:1635–1651.

Authors' Response 02-Nov-18

PP: We thank the reviewers for their comments, that have hopefully helped us to convey a clearer message in our paper. More importantly, the comments have helped us to address some omissions and inaccuracies.

1.) The authors state in the abstract that they investigated "the effects of both objective probability and subjective interpretation" of stop cues. In the discussion, however, they focus on subjective expectancy only. In addition, I believe that none of the contrast specified in the current version of the manuscript is suitable to investigate neural underpinnings of the objective probability of stop cues. The contrast "50% vs. 0%" comprises trials, in which stop-signals were subjectively expected to occur, as well as trials, in which stop-signals were subjectively expected not to occur. Therefore, this contrast of "objective probability" is contaminated by "subjective probability". Neural underpinnings of objective probability might be assessed by contrasting "50% vs. 0%" > "stop trials in which stop signal was subjectively expected". After having specified an appropriate contrast, the authors should focus in their discussion also on neural correlates of objective, cue-related activity.

PP: Thank you for the opportunity to clarify this point. Our aim involved analyzing data based on explicit information about subjective probability, as opposed to using contrasts defined only by objective cue-related probabilities (which will indeed show subjectivity-related effects). This has been clarified in the Abstract as follows: "To date, most studies of the brain areas underlying proactive inhibition have exclusively considered the objective predictive value of environmental cues, by varying the probability of stop-signals. However, by only taking into account the effect of different cues on brain activation, the subjective component of how those cues affect behavior is ignored. We used a modified stop-signal response task that includes a measurement for subjective expectation, **to investigate the effect of this subjective interpretation of cues**."

2.) Several aspects in the methods section, in particular in the subsection '2.5 Imaging' a described imprecisely.

a) Could the authors provide more information about which SPM version has been used?

PP: We apologize for leaving out the specific software version; now included in the methods section: "Data was processed using SPM8".

b) Was realignment really not performed in the preprocessing? It is not listed among the preprocessing steps. Since data was acquired in one single run with a duration of more than 18 minutes (683 volumes à 1600ms) (please cf. 2.3 Data acquisition), this is more than critical. Usually, a singly fMRI run has a duration of 5 to max. 10 minutes to minimize the probability of movement artifacts. Have the data been checked for movement artifacts? If so, the author should report that.

PP: We thank the reviewer for noticing this omission. As the reviewer rightly states, the realignment of fMRI data as means of motion correction is standard practice. We have done this during pre-processing of the data, but left this step out in our methods section by accident - it has been included.

c) Stimulus-related activation is described to be modeled as an epoch of 1000ms, yet specifications about cue-related activation (when not parametrically modulated) are lacking. Was cue-related activation (when not parametrically modulated) modeled using stick functions?

PP: Cue-related activation was modelled as epochs of 1500ms, we apologize for this omission.

d) Why was stimulus-related activation modeled as an epoch of 1000ms?

PP: The duration of the rising of the bar is 1000ms, and both the stimulus (stopping of the bar) and the response take place in that time window. However, as our article only focusses on activation during the cue period, we have removed this statement altogether to avoid confusion.

My major concern regarding the methodological approach, however, pertains to the clusterwise inference. As I gather from table 3 results were reported to be significant at a family-wise error corrected cluster level of $p < .05$. First of all, this needs to be reported in the methods section.

PP: The threshold is now also mentioned in the method section instead of only the figure caption.

More importantly, however, familywise error rate of 5% for clusterwise inference has been deemed invalid as they tend to result in high false positive error rates (Eklund et al., 2016, PNAS). The authors should accordingly use a more conservative approach to correct for multiple comparisons (i.e., voxelwise inference).

PP: We agree with the reviewer that a FWE clusterwise correction can lead to false positives when used without an a priori hypothesis using standard methods. however, the main result of our paper, the third contrast, is that we find activation in regions that we have associated with proactive inhibition previously. In addition, our results do not lie marginally above the threshold (with a threshold value of T = 3.4, and peak values in the striatum of T = 5.4. Furthermore, to calculate the appropriate threshold, we

have employed a tool created by TE Nichols (CorrClusth, http://www.sph.umich.edu/~nichols/JG5/CorrClusTh.m). We are therefore confident that are results are not based on a false positive. We have included a reference to this tool in the imaging method section 2.5:
"All brain activation maps will be thresholded at a family-wise error-corrected cluster level of $p < 0.05$, with cluster sizes determined using CorrClusTh (http://www.sph.umich.edu/~nichols/JG5/CorrClusTh.m)."

3.) Similar to the analyses of the fMRI data, the analyses of the behavioral are insufficiently described in the methods section. The authors vaguely describe in '2.4 Analyses' that "the effect of stop-signal expectation on accuracy and response times was assessed". In the results sections and in table 1, the authors then present a number of paired t-tests comparing accuracy between trials with '0% stop-signal probability' and trials with '>50% stop-signal probability' (why >50%? Stop signal probability has previously been reported to be 50%?!) for the following three conditions: 1) 'stop signal expected', 2) 'stop signal not expected', 3) 'no expectation indicated'. The authors should perform a Bonferroni correction for the number of tests performed (i.e., the three conditions) or use a repeated measures ANOVA).
PP: We apologize for the error and have replaced all instances of ">50%" by the correct "50%". We have clarified the comparisons as follows: "We compared performance between trials with 0% and 50% stop-probability cues for three different subsets of trials, defined by subjective expectation information: stop signal expected, stop signal not expected, and no expectation indicated," We have added a Bonferroni multiple-comparisons adjustment to our criterion for significance; this led to a minor change in the description of table 2 at the end of paragraph 3.1 – but no changes to the actual results.

In addition, in the second paragraph of '3.1 Behavior', the authors should describe the comparisons more precisely. If I understand correctly, in the first t-test, go RT was compared for 0% vs. 50% trials; in the second t-test, go RT was compared for 0% vs. 50% trials on which no stop signal was expected; and in the third t-test. Go RT was compared for 50% trials on which a stop signal was expected vs. those on which no stop signal was expected? More explicit guidance might be helpful for the reader and increase clarity of the manuscript.
PP: Please see our response above.

4.) As I gather from table 3 and Figure 4, in particular the putamen was associated with subjective expectation of a stop-signal, as revealed by with parametric modulation approach. In the text the authors report striatal activity to be associated with subjective expectation throughout the manuscript. I think it would be more specific to report putamen activity also in the text, since caudate seems not to be activated in the given contrast. In addition, the cluster covering putamen activity as revealed by the parametric modulation approach also covered right IFG – this should also be reported in the text.
PP: The reviewer is correct in his critique of our language use. We indeed have conflated the striatal subdivisions in the results and discussion section, even though activation was sometimes limited to the

putamen. We have made changes to the results and discussion to better reflect the specific activation pattern.

5.) The authors discuss the role of the right IFG. Hereby consideration must be given to regional specificity of the functional involvement of this rather large brain region (see e.g. the reply by Aron et al., 2014 TiCS and their criticism regarding the work of Adam Hampshire and David J. Sharp). In addition, when discussing rIFG involvement in attentional capture as reported by Sharp et al. (2010, PNAS), the authors should also mention that a number of studies using similar task reported opposing results (e.g., Boehler et al. 2011 PLoS ONE; Cai et al. 2011 PLoS ONE; Chikazoe et al. 2009 Cereb Cortex; Sebastian et al., 2016 Brain Struct Funct).
PP: We have added literature to give consideration to the regional specificity of the functional involvement of the IFG in the discussion ***(page 15)****:*

"Therefore, involvement of the rIFG in cue processing might be mediated by increased attention to these cues. Inherent to most tasks, it is impossible to completely distinguish attentive and inhibitory processes. Though Boehler et al. (2011) showed that the rIFG only responded to relevant stop cues and not to irrelevant stop cues appearing in a control block in which the participant was instructed to ignore the cues, pointing to a role in inhibition and not to infrequent cue detection, it might still be related to attentional engagement. It might also be that differential roles in attention and inhibition are regulated by distinct subareas within the rIFG or by differential network involvement (Sebastian et al., 2016, Verbruggen et al., 2010; Chikazoe et al., 2009a). Distinct functional roles in response inhibition have already been assigned to different IFG subregions (Cai & Leung, 2011; Chikazoe et al., 2009a)."

6.) The role of the middle frontal gyrus (DLPFC) in subjective expectation of a stop signal is barely discussed, although it is implicated by the parametric modulation approach.
We have now improved the discussion on the DLPFC effect and how it relates to the literature ***(page 16)****:*

"Activity in the mid frontal gyrus correlated with increasing response time, as an objective measure for stop-signal expectation. The dorsolateral prefrontal cortex (DLPFC), a prominent structure within the mid frontal gyrus, has been associated with response inhibition before (Hung et al., 2018; Hege et al., 2014; Luijten et al., 2014) and is thought to be involved in executive function, cognitive flexibility and planning. Specifically, this area was more active during conditional stopping -when participants only had to stop their responses in a specific context- than during simple stopping (Swann et al., 2012, Chikazoe et al., 2009b) and is also though to implement task rules (Aron et al., 2014)."

7.) The methods within the supplemental material are insufficiently described, too. The authors should at least provide information about the nature of the ROIs (were anatomically masks used?), about the procedure of signal extraction (was mean signal activity extracted or the activity within a peak voxel? Was percent signal change extracted, or beta or contrasts estimates?) and about the test that were performed (were that rm ANOVAS? Are the results corrected for multiple comparisons?).

PP: The supplemental materials are a replication of previous work (Vink et al, 2015), but perhaps indeed rely too much on said previous work to explain the underlying methods. The regions of interests where masks based on activation in previous research (Zandbelt et al. 2013). The replication used the same methodology, regions of interest and statistical analysis. We have added this information to the supplemental in order for it to be understandable on its own.

More information should also be provided for the supplemental figures (figures 5 and 6), at least the coordinates of the ROIs and the nature of the signal (i.e., percent signal change, beta or contrast estimates?).
PP: Information was added to the figure captions on the location of the ROIs and the nature of the signal.

Minors
i) I am sometimes confused about the tenses that the authors use. Everything pertaining to what was done in the present study should be written in the simple past tense. The authors should check the manuscript and correct the tense where applicable.
PP: We apologize for the inconsistent use of present/past tense. We have corrected this throughout the manuscript. Parts of the introduction leading up to the hypothesis are written in present tense, whereas the results of the study are now written consistently in simple past tense.

ii) '2.2 Stop-signal Anticipation task' – second sentence: " […] adapted from (Vink, Kaldewaij, et al., 2015)": please correct
PP: This has been corrected.

iii) '2.4 Analyses' – second last sentence: "[…] stop-signal reaction time, computed according to the integration (Logan & Cowan, 1984)": should be "integration method"
iv) Discussion, p. 13, last paragraph: "Activation has also been demonstrated to increase during cue-learning paradigms; being linked to the formation of stimulus-response associations": please correct
PP: This has been corrected.

v) Discussion, p. 14, first paragraph: "[…] and data by Seger (Seger, 2005)": should be "[…] and data by Seger (2005)"
PP: This has been corrected.

vi) References: The publication year of the reference by Sebastian et al. should be 2016 instead of 2015.
PP: This has been corrected.

vii) Table 3: The authors should double check the coordinates for the "Med. Frontal gyrus" (x=40 y=48 z=24) – or do they mean middle frontal gyrus instead of medial frontal gyrus?
PP: This has been corrected to 'mid frontal gyrus'.

2nd Editorial Decision 22-Nov-18

Dear Dr. Pas,

Your revised manuscript has been re-evaluated by one of the external reviewers as well as by the Section Editor, Dr. Chandan Vaidya and ourselves. We are pleased to inform you that we expect that it will be accepted for publication in EJN pending a few minor revisions. Once done we will send it off to the production editors.

Although the reviewers indicated that your manuscript has been substantially improved, they continue to raise concerns that need to be addressed more effectively.

- Please proof read before final acceptance as suggested by the reviewer and we request that you incorporate the suggestion that limitations should include acknowledgement that multiple factors contribute to response slowing.
- Please include the figure legends into the main text (at the end).
- You have not include 'Acknowledgements', was that an oversight?

When revising the manuscript, please embolden or underline major changes to the text so they are easily identifiable and DO NOT leave 'track change' formatting marks in your paper. At this stage, please provide text and a figure file for the Graphical Abstract. When carrying out your revisions please refer to the checklist below and visit the EJN author guidelines at www.ejneuroscience.org

When finalised, please upload your complete revised manuscript onto the website as a Word (.doc or .docx), or .rtf file. Please also ensure that a complete set of tables and figures is included as separate files, even if these have not changed from the originals. At this stage it is necessary to provide high resolution figures. Please see important instructions below.

Please go into https://mc.manuscriptcentral.com/ejn - Author Centre - manuscripts with decisions where you will find a 'create a revision' link under 'actions'. We ask that you please indicate the way in which you have responded to the points raised by the Editors and Reviewers in a letter. Please upload this response letter as a separate Word (.doc) file using the file designation "Authors' Response to Reviewers" when uploading your manuscript files. Please DO NOT submit your revised manuscript as a new one. Also, please note that only the Author who submitted the original version of the manuscript should submit a revised version.

If you are able to respond fully to the points raised, we shall be pleased to receive a revision of your paper within 30 days.

Thank you for submitting your work to EJN.

Best wishes,

Paul Bolam & John Foxe
co-Editors in Chief, EJN

Reviews:

EJN European Journal of Neuroscience FENS Federation of European Neuroscience Societies

Reviewer: 1

Comments to the Author
The revised manuscript (including the supplemental material) by Pas and colleagues has improved a lot with regard to clarity and detailed and accurate data. I just have some minor remarks that might further improve the quality of the manuscript.

1) I agree with the authors that proactive inhibition is on average reflected in response time slowing. On a trial-by-trial basis, however, I would argue that other factors might also influence reaction time variability. If we take for example the drift decision model, reaction time is composed by sensory processing of the stimulus, the quality of information processing, the response caution, the a priori bias for a specific response and finally response execution. All these processes are likely influenced by other processes, such as speed-accuracy tradeoff, attentional processes etc. The authors should thus acknowledge at least in their limitation section that RT variability does not exclusively depend on subjective expectation of a stop-signal but might also be influenced by other factors.

2) The authors should once more double check the use of tenses. I believe that in particular some paragraphs in the introduction need to be edited, e.g. introduction, p.10:
- "25 healthy volunteers will perform a modified delayed-response stop-signal anticipation task…" --> should be "… volunteers performed…"
- "In this way, we are able to investigate proactive inhibition…" --> should be "we were able to investigate…"
- "Importantly, we focus on the cue period that occurs 1000 to 2000 ms prior to the onset of the stimulus" --> should be "Importantly, we focused…)
- "We hypothesize that by modelling activation during the cue period" --> should be "We hypothesized that…"

3) Introduction, p.10: "25 healthy volunteers" --> should be "Twenty-five healthy volunteers …"

4) Materials and methods, p.11: "Twenty-five volunteers (Age M=21.6 years, …" --> should be "(age M=21.6 years,…"

Authors' Response 26-Nov-18

---

1) I agree with the authors that proactive inhibition is on average reflected in response time slowing. On a trial-by-trial basis, however, I would argue that other factors might also influence reaction time variability. If we take for example the drift decision model, reaction time is composed by sensory processing of the stimulus, the quality of information processing, the response caution, the a priori bias for a specific response and finally response execution. All these processes are likely influenced by other processes, such as speed-accuracy tradeoff, attentional processes etc. The authors should thus acknowledge at least in their limitation section that RT variability does not exclusively depend on subjective expectation of a stop-signal but might also be influenced by other factors.

PP: We agree with the reviewer in his critique of our limitation section, and have added the following paragraph at the end:

Page 17: "Our results are based on the assumption that the slowing down of responses can, at least partially, be explained by the expectation people form based on the cues. However, a multitude of factors come into play on each trial that can affect response times, ranging from the effectiveness and speed of sensory processing, quality of information processing or a potential a priori bias for a specific response. Sequential-sampling models like the diffusion model describe decision making as a process of noisy accumulation of evidence from a stimulus (Ratcliff *et al.,* 2016). Reaction time variability can therefore not be claimed to exclusively depend on the subjective expectation of a stop signal, and there is still noise left unexplained."

2) The authors should once more double check the use of tenses. I believe that in particular some paragraphs in the introduction need to be edited, e.g. introduction, p.10:
- "25 healthy volunteers will perform a modified delayed-response stop-signal anticipation task…" --> should be "… volunteers performed…"
- "In this way, we are able to investigate proactive inhibition…" --> should be "we were able to investigate…"
- "Importantly, we focus on the cue period that occurs 1000 to 2000 ms prior to the onset of the stimulus" --> should be "Importantly, we focused…)
- "We hypothesize that by modelling activation during the cue period" --> should be "We hypothesized that…"

3) Introduction, p.10: "25 healthy volunteers" --> should be "Twenty-five healthy volunteers …"

4) Materials and methods, p.11: "Twenty-five volunteers (Age M=21.6 years, …" --> should be "(age M=21.6 years,…"

PP: We thank the reviewer of looking at our manuscript so thoroughly, and have made the changes that were pointed out.
